# Intercontinental transmission and local demographic expansion of SARS-CoV-2

**DOI:** 10.1017/S0950268821000777

**Published:** 2021-04-13

**Authors:** Hong-yin Hu, Fang Yan, Jia-ming Zhu, Alex Plimo Karuno, Wei-wei Zhou

**Affiliations:** 1 State Key Laboratory of Grassland Agro-Ecosystem, Institute of Innovation Ecology & School of Life Sciences, Lanzhou University, Lanzhou, China; 2 College of Life Science, Yunnan University, Kunming 650500, China; 3 State Key Laboratory of Genetic Resources and Evolution, Kunming Institute of Zoology, Chinese Academy of Sciences, Kunming, 650223, China

**Keywords:** COVID-19, demographic expansions, epidemic, genome, intercontinental transmission

## Abstract

The global outbreak of coronavirus disease 2019 (COVID-19) is greatly threatening the public health in the world. We reconstructed global transmissions and potential demographic expansions of severe acute respiratory syndrome coronavirus 2 based on genomic information. We found that intercontinental transmissions were rare in January and early February but drastically increased since late February. After world-wide implements of travel restrictions, the transmission frequencies decreased to a low level in April. We identified a total of 88 potential demographic expansions over the world based on the star-radiative networks and 75 of them were found in Europe and North America. The expansion numbers peaked in March and quickly dropped since April. These findings are highly concordant with epidemic reports and modelling results and highlight the significance of quarantine validity on the global spread of COVID-19. Our analyses indicate that the travel restrictions and social distancing measures are effective in containing the spread of COVID-19.

## Introduction

The outbreak of coronavirus disease 2019 (COVID-19) [1] caused by severe acute respiratory syndrome coronavirus 2 (SARS-CoV-2) [2] has been and is still threatening global public health. The origin of SARS-CoV-2 is still under debate. It is difficult to find the root cause of the disease and different approaches yield contrasting results [3]. Meanwhile, two studies based on network analyses identified ancestral haplogroup by using sequences of coronavirus from bats as an outgroup [4, 5]. However, the reliability of this result is under debate [6–8]. After an outbreak in east Asia, COVID-19 dramatically spread worldwide. The first cases were confirmed in Europe, North America and Oceania in January 2020. In February, the cases were officially reported in all continents. Until May 2020, the officially confirmed cases were reported in more than 200 countries. Up to 1 July 2020, the world-wide outbreak of COVID-19 had caused more than 510 thousand deaths (https://coronavirus.jhu.edu/map.html). Studies based on epidemic models evaluated the transmissions and expansions of SARS-CoV-2 in the world [9–14], suggesting the likely complex demographic dynamics. However, the direct evidence from population genomic data is lacking. This is critical to trace transmission events and identify community-based outbreaks based on the demographic expansions [15, 16]. After the outbreak of COVID-19, a large number of viral genomes were sequenced and uploaded to the public database (https://www.gisaid.org/). These open resources provide us a chance to examine evolutionary history of the SARS-CoV-2. We can trace the intercontinental transmission and potential demographic expansions against timescales and accompanying travel restrictions in the world. In this study, we evaluated the impact of travel restrictions on the spread of COVID-19 based on genomic information. Meanwhile, we checked if the strong social distancing measures are effective in reducing local demographic expansions of virus.

## Materials and methods

### Sequences selection

To reconstruct the evolutionary history of SARS-CoV-2, we downloaded 31 820 full-length genome sequences on 26 May 2020. The sequences were downloaded from the Global Initiative on Sharing Avian Influenza Data (GISAID) (Table S1). We also collected the sampling time of the sequences. As the random sequencing errors could generate a large amount of single site mutation, which would affect the accurate definition of the haplotype and network, we removed all low-quality sequences following the reference [17]. We filtered the data based on mutation density and numbers of unknown bases (N), degenerate bases and gaps. Mutation density was defined by mutations/slide window size (20nt). The gaps were identified by comparing with reference sequence (MN908947). The unknown bases should be less than 15, the degenerate bases should be less than 50 and the gaps should be less than three. If a sequence contained high mutation density regions (mutation density larger than or equal to 0.25), it would also be treated as low quality.

### Construction of phylogenetic tree and network

The genome sequences were aligned using MAFFT [18]. Haplotypes were determined using the R package ‘pegas’ [19]. We used NETWORK 4.10 to build a median-joining network [20]. The phylogenetic tree was constructed by using IQtree [21]. Support values were estimated by using ultrafast bootstrap approximation [22, 23] with 1000 bootstrap replicates. Haplotype diversity and nucleotide diversity were calculated using the R package ‘pegas’ [19].

### Transmission pattern reconstruction

Based on the sampling date of the genome sequences, we obtained the potential origin time for each haplotype. We set the earliest sampling date as origin time of the haplotype and the sampling dates of sequences in other continents as transmission time. We corrected origin time and place of haplotypes based on the epidemic information, especially the travelling history of the first few confirmed cases of each country. For some haplotypes, the first reported sequences were isolated from patients who had an international travelling history. Therefore, we corrected the origin place as where they may be infected. In total, the origin place of eight haplotypes were corrected (H10, H121, H3196, H2714, H2354, H179, H1565 and H1035). We assumed that the same haplotype or two directly connected haplotypes in the network found in the different continents represented one intercontinental transmission. The links with more than 10 mutations were not involved in the analyses, as it may be caused by sequencing errors.

### Local demographic expansion

The rapid population expansion of the virus through transmission will lead to the production of the numerous haplotypes with short step (one or two mutations) from one central haplotype [24]. Except seriously sampling biases, the frequency of these haplotypes should be lower than the central haplotype. This local demographic expansion from the potential virus outbreak could be identified by the star-like phylogeny of the haplotypes in the network [24]. We set haplotypes that linked with 10 or more haplotypes in the network as central haplotype. Then we extracted the haplotypes directly connected with the central haplotypes. The samples of these haplotypes together with the central haplotype were used to define local demographic expansion because of the outbreaks of SARS-CoV-2. We examined the distributions of each expansion in the continent and the total occurrence frequency of expansions. Analyses were repeated under different thresholds to see if we can get similar patterns. We also assigned sequences to clades following the approach of Rambaut *et al*. [25]. Then we checked the frequency of outbreaks for each clade.

## Results

### Genome sequence variation and network construction

**Fig. 1.**
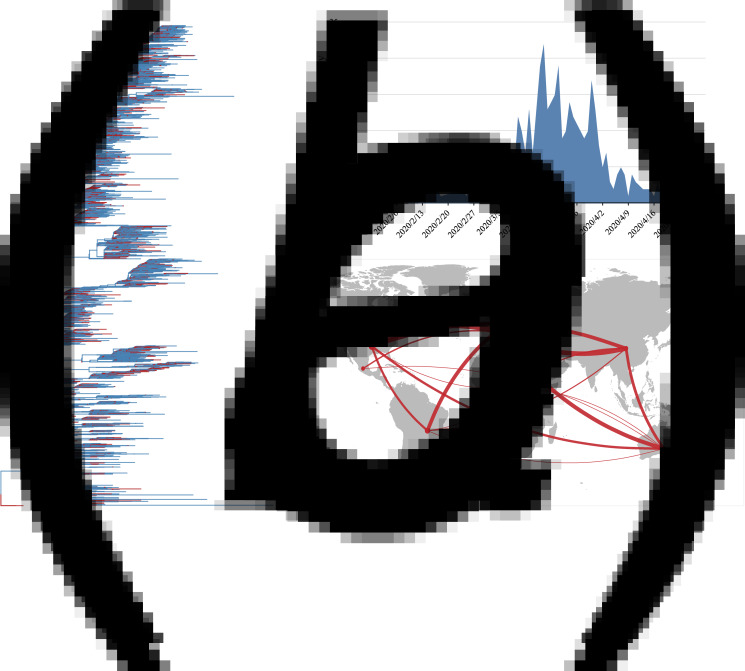
(a) A phylogeny tree based on SARS-CoV-2 genomes by IQtree. Haplotypes associated with the local demographic expansions identified by network analyses are indicated in red. (b) Intercontinental transmissions against timescale. x-axis stands for time and y-axis stands for number of transmissions. (c) The illustrated intercontinental transmissions between different continents and the line thickness correspond to transmission frequencies.

In total, we downloaded 31 820 full-length genome sequences and 14 206 sequences passed the quality controls. After trimming the ends, the alignment we used was 29 182 bps. The alignment included 6275 variable sites, in which 2757 are potential phylogenetic informative sites. In addition, we identified a total of 7177 haplotypes from all the reported genomes (Table S1). We constructed the phylogenetic tree (Fig. 1a) and the network based on sequences of haplotypes. The overall nucleotide diversity was 0.000098 and the haplotype diversity was 0.996.

### Global transmission pattern of COVID-19

When a haplotype or two directly connected haplotypes on the network were found in different continents, one intercontinental transmission was defined. In total, we identified 412 intercontinental transmissions (Table S2). The transmissions occurred rarely in January and February, but drastically increased since the end of February (Fig. 1b). The transmission frequencies peaked in mid-March, which was about 10 times higher than those in January. Since April, the transmission frequencies dropped down to a low level as in January when travel restrictions were implemented in the world.

### Local demographic expansion

**Fig. 2.**
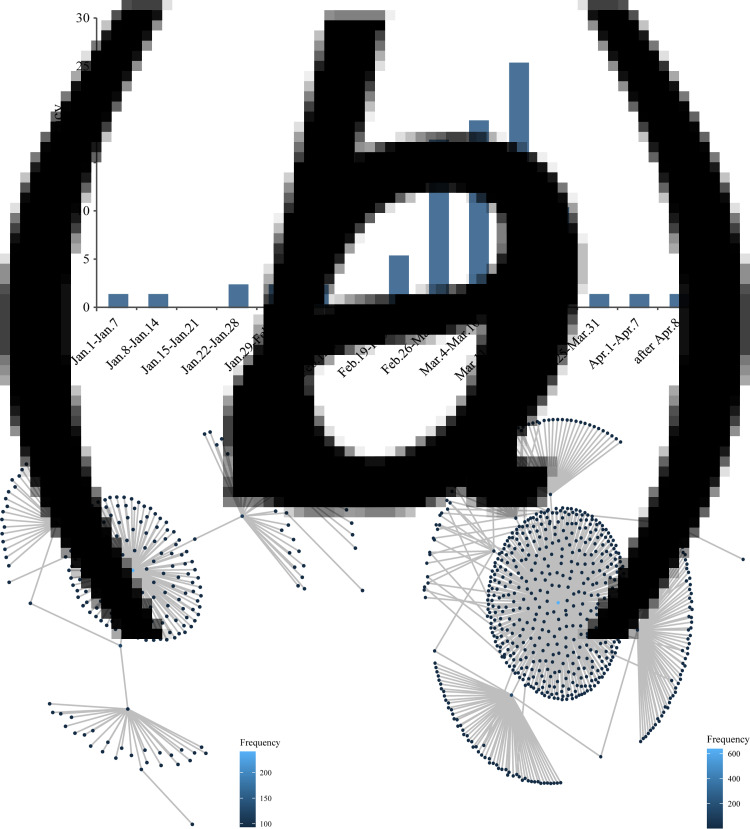
(a) The demographic expansions against timescale. x-axis indicates time and y-axis shows the number of expansions. (b) Examples of the star-radiative structures in the median-joining subnetwork of the SARS-CoV-2 genomes. Colours stand for haplotype frequency.

Rapid population expansion because of frequent transmissions of virus will lead to the fixations of rare and random mutation due to founder effect [15, 26]. This will result in the star-phylogeny network, in which rare haplotypes with short mutation are connected to a central haplotype with high frequency [24]. We defined a subnetwork with 10 or more haplotypes with few mutations connected to one high-frequency central haplotype as one potential expansion. In total, we identified 88 potential demographic expansions of COVID-19 (Table S3 and Fig. 2a) by this approach. Temporal pattern of the expansion, which yielded from the earliest sampling time of central haplotypes, indicated that most expansions emerged since the end of February. Six expansions occurred before February, 18 in February, 61 in March and 2 since April. For the 18 ones in February, 16 of them took place after 20 February. Following the clade assignment, we identified 23 expansions in clade 19A, 13 ones in clade 19B, 25 ones in clade 20A, 10 ones in clade 20B and 17 ones in clade 20C (Tables S3 and S4). The expansion frequencies may change when different thresholds were used to define each expansion, but the total trend remains similar (Table S5).

## Discussion and conclusion

Based on the 14 206 sequences that passed the filtering, we identified 412 intercontinental transmissions. Our analyses suggested that the intercontinental transmissions occurred at a lower frequency in January and February (Fig. 1b). However, since the end of February, such intercontinental transmissions increased drastically at a high frequency (Fig. 1b). Most identified transmissions are related with Europe and North America. The intercontinental transmissions started to decrease since mid-March, which is roughly concordant with the time that large-scale travel bans started to be implemented in the world. The frequency returned to the low level since April. Undoubtedly these transmissions only comprised a part of the actual transmissions. Considering the incubation period, the transmission times we estimated may be later than the real time, but the general pattern of transmissions was not impacted by such bias. It should be noted that although travel bans reduced the transmission frequencies, these policies failed to prevent the continuing epidemic of COVID-19. A recent study presented similar patterns [27]. The European clade originated from east Asian clade. However, the subsequent worldwide spread since March 2020 were mainly related to the European clade [27]. As suggested by the epidemic model fitting [9], very few transmissions may have led to severe epidemics and secondary outbreaks when social distancing measures or strict community quarantine are lacking.

This study further identified 88 potential demographic expansions of SARS-CoV-2. Most expansions were reported in Europe and North America. The temporal pattern indicated that multiple expansions occurred when quarantine policies to reduce social contacts had not been implemented [28]. The local expansions occurred at a lower frequency as the intercontinental transmissions in January and February (Fig. 2a). However, since the end of February, the frequency of expansions drastically increased and peaked in March. After strong social distancing measures were strictly imposed [28], the number of expansions quickly dropped (Fig. 2a and Table S3). Only two expansions were identified since April. Similar patterns were observed when applying different thresholds (Table S5). This is concordant with other studies [13] and the trends of daily new cases (https://coronavirus.jhu.edu/map.html). For example, in Europe and North America, daily new cases peaked in mid-March or early April, then the curve reached a plateau or started to drop in most countries (https://coronavirus.jhu.edu/map.html).

Overall, the present study examined intercontinental transmission and local demographic expansion of the SARS-CoV-2 in the world based on a big dataset of the genomes. These findings inferred directly from the genome sequences are largely concordant with the epidemic reports and epidemic modelling [11, 12]. Our network analyses illustrated history and relationships of populations without any prior assumption on demographic histories. These results can be used as guidance in subsequent modelling and other studies. Both sample numbers and sampling bias may affect our estimations. For example, transmissions and expansions could not be estimated based on our methods when very few sequences are available. Dense samplings will identify more transmissions and expansion events. But our conclusions are not impacted by such bias, as the genome sequences were continuously increasing in April and May (Table S1). In this way, the decrease of frequency of international transmissions and number of expansions since April could not be an artefact because of fewer samples. Meanwhile, our results are concordant with other studies [27], which indicate the results are reliable. Despite these caveats, we provided the genomic evidence for quarantine validity on the spread of COVID-19 between continents across the world.

## Data Availability

The data that support the findings of this study are available from GISAID (https://www.gisaid.org/).

## Supplementary material

For supplementary material accompanying this paper visit http://dx.doi.org/10.1017/S0950268821000777. click here to view supplementary material
